# Research update on cell membrane camouflaged nanoparticles for cancer therapy

**DOI:** 10.3389/fbioe.2022.944518

**Published:** 2022-08-05

**Authors:** Chengfang Wang, Size Wu

**Affiliations:** Department of Ultrasound, The First Affiliated Hospital of Hainan Medical University, Haikou, China

**Keywords:** tumor therapy, biomimetic, cell membrane-coated nanoparticle, immune evasion, homotypic targeting

## Abstract

Cell membrane-camouflaged biomimetic functionalization of nanoparticles has emerged as a promising strategy for cancer theranostics. These cell membranes used for camouflaging are generally isolated from natural or engineered erythrocytes, neutrophils, macrophages, T lymphatic cells, stem cells, and cancer cells. The camouflaging strategy of coating nanoparticles with cell membranes allows for tumor homotypic targeting through self-recognition as source cells, immune evasion, and a prolonged blood circulation time, thereby improving the effective payload delivery and tumor therapy. More so, some engineered cell membranes with functionalized peptides, proteins and moieties on membrane surface can be transferred for therapy in the same time. In this review, we summarize the latest research on various types of cell membrane-camouflaged nanoparticles aimed at anti-cancer therapy, focusing on the biological advantages of different cell membranes, constitutions of nanoparticles, fabrication processes, key findings, potential therapies, and discuss the major challenges and future opportunities.

## 1 Introduction

Cancer has become one of the leading causes of disease-related death in the world with the growth of the aging population (Schilsky et al., 2020; Sung et al., 2021). The treatment of cancer has been changing and patients’ options now include surgical treatment, chemotherapy, radiotherapy, interventional treatment, and immune checkpoint inhibitor-based immunotherapy, among others (Schilsky et al., 2020; Seligson et al., 2021). Despite the continued progress that has been made, challenges remain to a certain extent as the therapeutic effects show only marginal increases, and adverse effects have not been eliminated or substantially decreased, thus often resulting in poor therapeutic outcomes (Bonsignore et al., 2021; Seligson et al., 2021).

To solve these issues, several measures have been adopted, such as using optimization of drugs’ size, shape, and surface charge, controlled release, targeted delivery, and a biomimetic nanoarchitecture to improve cancer treatment (Wu et al., 2021a; Castro et al., 2021; Jha et al., 2021). Targeted delivery comprises active and passive delivery. To reach its target in the tumor, the drug must overcome several difficulties after systematic administration, such as immune clearance, the barrier of the capillary endothelium, and the impediment of the extracellular matrix (Attia et al., 2019; Castro et al., 2021). In active targeting, a cell-specific ligand with the ability to bind to specific receptors overexpressed on tumors is conjugated to the therapeutic cargo; and in passive targeting, the therapeutic payload reaches the desire site by enhanced permeability and retention (EPR) effect (Attia et al., 2019; Dash et al., 2020). The ligand works when it nears or arrives at the target site, taking advantage of its increased affinity for the target.

Biomimetics was introduced to overcome associated challenges, and biomimetic nanoparticles (NPs) were thus fabricated. Biomimetic NPs consist of either material-based core NPs coated the biomimetic membrane or biomimetic membrane itself acting as a therapeutic cargo carrier (Attia et al., 2019; Dash et al., 2020; Jha et al., 2021). Biomimetic NPs camouflaged with cell membrane can exhibit cell-like behaviors, potentially affording them a prolonged circulation time (Hu et al., 2011; Liu et al., 2018; Xuan et al., 2018; Bidkar et al., 2019; Huang et al., 2019; Lee et al., 2019; Wang et al., 2020a; Wang et al., 2020b; Wang et al., 2020c; Dash et al., 2020; Li and Zhang, 2020; Peng et al., 2020; Wu et al., 2021b), immune escape (Pitchaimani et al., 2018; Cao et al., 2020; Wang et al., 2021a; Liu et al., 2021; Zhang et al., 2021), and/or increased targeting abilities (Parodi et al., 2013; Gao et al., 2016; Zhang et al., 2017; Pitchaimani et al., 2018; Yang et al., 2018; Bidkar et al., 2019; Cao et al., 2020; Wang et al., 2020c; Gong et al., 2020; Li et al., 2020; Poudel et al., 2020; Wu et al., 2020; Chen et al., 2021a; Li et al., 2021a; Wang et al., 2021a; Zhao et al., 2021a; Wu et al., 2021c; Fan et al., 2021; Liu et al., 2021; Shen et al., 2021; Zhang et al., 2021) on their intravenous administration.

Cell membrane-camouflaged NPs normally comprise a “core–shell” structure, in which the therapeutic payload loaded NP is the core, and a thin layer of cellular plasma membrane coating over the core NP is the shell. The core NP carries the payload that needs to be delivered to the desired site. Membranes obtained from different source cells are isolated and formed membrane vesicles through a combination of procedures. The obtained membrane vesicles are then coated onto the core NPs by co-extruding the membrane vesicles with the core NPs or a combination of sonication and extrusion (Attia et al., 2019; Dash et al., 2020; Castro et al., 2021; Jha et al., 2021). There are also other techniques such a microfluidic electroporation to form the shell (Jha et al., 2021).

**FIGURE 1:**
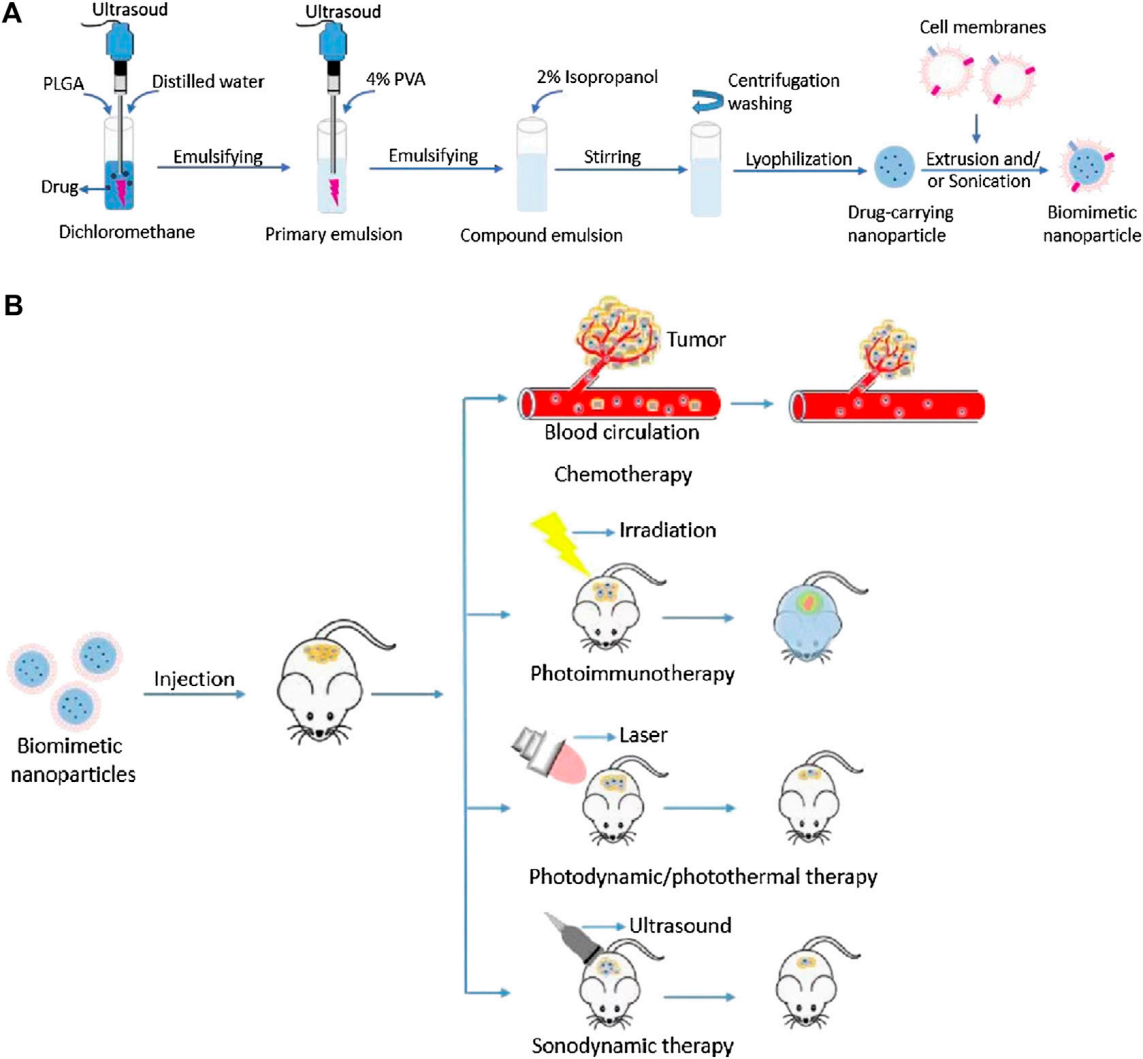
**(A)** Schematic illustration of fabrication processes of cell membrane-camouflaged drug loaded PLGA nanoparticles; **(B)** Schematic illustration of the potential anticancer therapies of cell membrane-camouflaged biomimetic nanoparticles. PLGA: Poly (lactic-co-glycolic acid).

The shell derived from the source cell shares the same innate properties of self-recognition. Cell membranes provide innate transmembrane proteins and moieties with little loss in functionality during therapeutic agents’ formulation for delivery, such as membrane-bound antigens that are essential for immune evasion and targeting (Attia et al., 2019; Dash et al., 2020; Castro et al., 2021; Jha et al., 2021). The cell membrane coating acts as a medium to functionalize synthetic NPs, making for a suitable delivery vehicle in various biomedical applications (Dash et al., 2020; Jha et al., 2021). The choice of cell membrane depends on the target site and desired therapeutic aims. The preferential delivery and retention of membrane-coated NPs deep in the tumor improve the therapeutic efficacy of antitumor and reduce systemic toxicity (Attia et al., 2019; Dash et al., 2020; Castro et al., 2021; Jha et al., 2021). Figure 1A illustrates the fabrication processes of cell membrane-camouflaged therapeutic payload loaded NPs; and Figure 1B illustrates the potential anticancer therapies of biomimetic NPs.

The first to be developed biomimetic NPs were red blood cell (RBC) membrane-camouflaged NPs (Hu et al., 2011; Huang et al., 2019; Castro et al., 2021). Since the successful proof-of-concept study that using cloaking NPs with cellular membranes isolated from freshly harvested erythrocytes and transferring bioactive cellular components to the surface of synthetic materials in order to confer unique functions not otherwise attainable through other bioconjugation techniques a decade ago, many source cells have been used for the fabrication of cellular membrane-camouflaged NPs (Table 1), including erythrocytes (Hu et al., 2011; Liu et al., 2018; Xuan et al., 2018; Huang et al., 2019; Lee et al., 2019; Wang et al., 2020a; Li and Zhang, 2020; Peng et al., 2020; Wu et al., 2021b), neutrophils (Wang et al., 2021a; Zhang et al., 2021), macrophages (Parodi et al., 2013; Cao et al., 2020; Poudel et al., 2020), natural killer (NK) cells (Pitchaimani et al., 2018), cytotoxic T cells (Zhang et al., 2017), stem cells (Gao et al., 2016; Yang et al., 2018), platelets (Wu et al., 2020; Li et al., 2021a), and cancer cells (Li et al., 2020; Chen et al., 2021a; Zhao et al., 2021a; Fan et al., 2021; Shen et al., 2021).

**TABLE 1:** Summary of different source cells for membrane camouflaged nanoparticles.

Source cell	References	Author
Red blood cell	(Hu et al., 2011; Pei et al., 2018; Wan et al., 2018; Xuan et al., 2018; Bidkar et al., 2019; Huang et al., 2019; Lee et al., 2019; Wang et al., 2020a; Wang et al., 2020c; Li and Zhang, 2020; Peng et al., 2020; Zhai et al., 2020; Wu et al., 2021b; Gao et al., 2021)	Hu et al., Wang et al., Li et al., Lee et al., Wu et al., Huang et al., Xuan et al., Peng et al., Bidkar et al., Wang et al., Wan et al., Zhai et al., Pei et al., Gao et al
Platelet	(Xu et al., 2018; Chen et al., 2019; Wang et al., 2019; Jiang et al., 2020b; Wu et al., 2020; Li et al., 2021a; Lyu et al., 2021)	Wu et al., Li et al., Chen et al., Wang et al., Jiang et al., Lyu et al., Xu et al
Neutrophil	(Wang et al., 2021a; Zhao et al., 2021b; Zhang et al., 2021)	Zhang et al., Wang et al., Zhao et al
Cancer cell	(Jiang et al., 2020a; Wang et al., 2020b; Wang et al., 2020d; Li et al., 2020; Chen et al., 2021a; Zhao et al., 2021a; Fan et al., 2021; Jin et al., 2021; Liu et al., 2021; Shen et al., 2021; Xu et al., 2021)	Wang et al., Liu et al., Li et al., Chen et al., Zhao et al., Shen et al., Fan et al., Jiang et al., Wang et al., Jin et al., Xu et al
Nature killer cell	Pitchaimani et al. (2018)	Pitchaimani et al
Macrophage	(Parodi et al., 2013; Evangelopoulos et al., 2016; Palomba et al., 2016; Molinaro et al., 2018; Bhattacharyya and Ghosh, 2020; Cao et al., 2020; Gong et al., 2020; Ji et al., 2020; Poudel et al., 2020; Xia et al., 2020; Chen et al., 2021b)	Parodi et al., Cao et al., Poudel et al., Gong et al., Evangelopoulos et al., Bhattacharyya et al., Chen et al., Xia et al., Molinaro et al., Palomba et al., Ji et al
Stem cell	(Gao et al., 2016; Yang et al., 2018; Li et al., 2021b; Mu et al., 2021; Zinger et al., 2021)	Gao et al., Yang et al., Zinger et al., Mu et al., Li et al
T-lymphocyte	(Evangelopoulos et al., 2016; Palomba et al., 2016; Zhang et al., 2017; Molinaro et al., 2018; Ma et al., 2020)	Zhang et al., Ma et al., Evangelopoulos et al., Molinaro et al., Palomba et al
Erythrocyte-platelet	Liu et al. (2018)	Liu et al
Platelet-Tumor cell	Wu et al. (2021c)	Wu et al
Macrophage-cancer cell	(Gong et al., 2020; Ji et al., 2020)	Gong et al., Ji et al
Erythrocyte-cancer cell	(Jiang et al., 2019; Xiong et al., 2021)	Xiong et al., Jiang et al
143B epithelioid cell-RAW264.7 cell	Cai et al. (2022)	Cai et al

During the past decade, great advances have been made, membranes used for camouflaging from cell membranes derived from single source cell to hybrid cell membranes derived from several source cells, from the use of the innate properties of cell membranes to the combination use of both the innate and acquired properties of cell membranes from engineered source cells, or with modification of cell membrane surface charge; with different methods for isolation of cell membranes and coating (Zhang et al., 2017; Yang et al., 2018; Wang et al., 2020b; Li et al., 2020; Wang et al., 2021a). Customizable exosome‐like lipid nanovesicles have been engineered by integrating membrane proteins that are unbiasedly sourced from human pluripotent stem‐cell‐derived neurons for next‐generation functionalized theranostics; in the process both endogenous and genetically engineered cell‐derived proteins can be transferred effectively without disruption of physicochemical properties (Zinger et al., 2021). Genetically engineered chimeric antigen receptors (CARs) T lymphatic cell and natural T lymphatic cell that extracted from human T cells enriched from peripheral blood mononuclear cells have been obtained, the CAR-T cells could recognize GPC3 expressed on the surface of hepatocellular carcinoma cells, enhancing targeting ability, and the CAR T cells could eliminate cancer cells by single-chain variable region (ScFv) on the cell membranes of CAR-T cells, in a non-major histocompatibility complex-restricted way (Ma et al., 2020). Wild-type cancer cells (wild-type B16-F10 murine melanoma cells) can be genetically engineered to express a co-stimulatory marker that enables them to directly present their own antigens to the immune system under an immunostimulatory context. NPs cloaked with cell membranes from these engineering modified cancer cells are able to elicit anticancer immunity *in vivo* while sparing the need for conventional cell-mediated antigen presentation (Jiang et al., 2020a). These present a great promising for the development and antitumor application of cell membrane-camouflaged NPs. Meanwhile, various potential side effects that can be caused by cell membranes for NPs have been studied. The response of the complement and to the cascade of reactions that occurs on the surfaces of injected NPs and that generates active components with various effector functions has been investigated. Cell membranes coated NPs and biological systems are crucial to predict and interpret their biodistribution, targeting, and efficacy, and thus need to design more effective therapeutic payload delivery systems. NPs are coated by a protein corona after intravenous injection. This confers a new biological identity on the NPs that largely determines their biological fate. In this context, the formation of a protein corona and of its effect on NPs’ function have also been studied, which provides reference for the design of more effective therapeutic carriers (Corbo et al., 2016; Liu et al., 2021). To probe potential side effects that can be caused by syngeneic and xenogeneic membranes (i.e., murine and human) applied to synthetic NPs, a comprehensive study was conducted. The results showed that the source of membrane is critical in inhibiting cellular internalization and rapid clearance of the NPs, although less so in triggering an acute inflammatory response (Evangelopoulos et al., 2016). This implies that to design new therapeutics, attention should be paid to not elicit an immune activation. Meanwhile, inspired by the popularity of cell membrane-camouflaging, diverse novel core NPs were fabricated using top-down or bottom-up fabrication approaches for diverse aims of PTT, PDT, SDT, immunological therapy, starvation therapy, imaging, diagnosis, etc (Zhang et al., 2017; Chen et al., 2019; Lee et al., 2019; Bhattacharyya and Ghosh, 2020; Wang et al., 2020b; Li et al., 2020; Poudel et al., 2020; Liu et al., 2021; Mu et al., 2021; Xiong et al., 2021; Zhang et al., 2021; Zinger et al., 2021).

**FIGURE 2:**
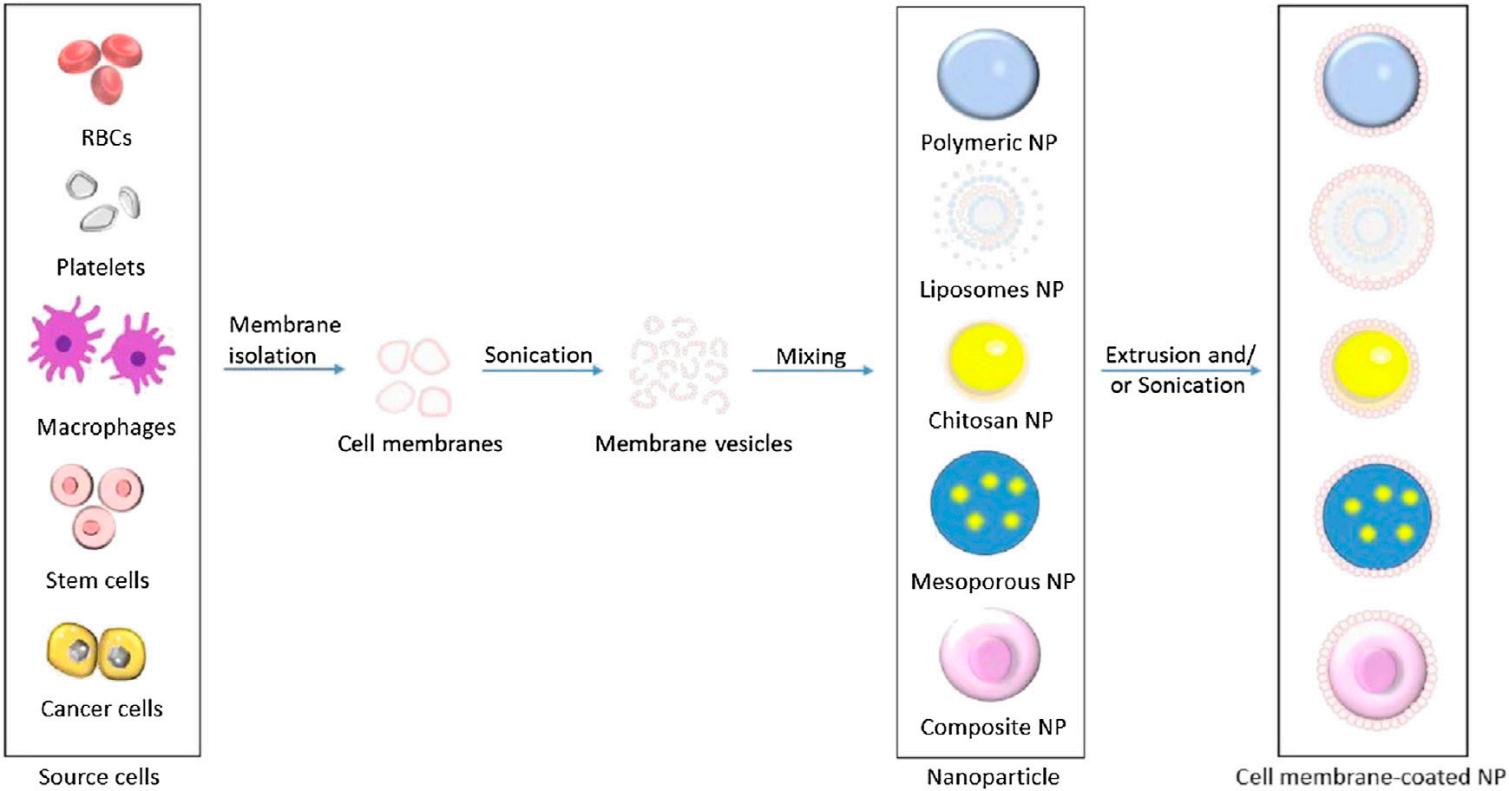
Schematic illustration of processes of isolating cell membranes and making cell membrane vesicles from different source cells for camouflaging different core nanoparticles.

Figure 2 illustrates the processes of isolating cell membranes from different source cells for NPs camouflaging. This review summarizes the recent cutting-edge research on different types of cell membrane-camouflaged NPs, related source cell properties, formulation processes, important findings, and potential applications in cancer therapy.

## 2 Components and development of cell membrane-camouflaged NPs

A cell membrane-camouflaged NP typically consists of an NP loaded with a therapeutic payload in the center (core) and a thin layer of cellular plasma membrane on the outside (shell), forming a core–shell structure (Dash et al., 2020; Jha et al., 2021). The development of cell membrane-camouflaged NPs comprises three processes: isolating cell membranes from the source cells, designing the core NP, and fusing cell membranes with the core NP to form a core–shell structure (Figure 1). All preparation processes are important in the development of cell membrane-cloaked NPs, including derivation of the lipid bilayer of the plasma membrane from source cells. Cell membranes that separate the cell cytoplasm from the outer environment are phospholipid bilayer semi-permeable structures with embedded and bound proteins and other motifs. The isolation of plasma membranes from source cells mainly involves a combination of hypotonic lysis, mechanical membrane fragmentation, and emptying the intracellular contents through differential centrifugation (Parodi et al., 2013; Gao et al., 2016; Zhang et al., 2017; Pitchaimani et al., 2018; Yang et al., 2018; Cao et al., 2020; Gong et al., 2020; Li et al., 2020; Poudel et al., 2020; Wu et al., 2020; Chen et al., 2021a; Li et al., 2021a; Wang et al., 2021a; Zhao et al., 2021a; Wu et al., 2021c; Fan et al., 2021; Liu et al., 2021; Shen et al., 2021; Zhang et al., 2021). Generally, cell membrane vesicles used for core NPs coating are obtained using a combination of hypotonic lysis buffer to lyse cells or the freeze–thaw method to obtain cell membranes; mechanical disruption of cell membranes using a dounce homogenizer, sonication, and/or extrusion, and finally, differential ultracentrifugation to obtain membrane vesicles (Parodi et al., 2013; Gao et al., 2016; Zhang et al., 2017; Liu et al., 2018; Pitchaimani et al., 2018; Xuan et al., 2018; Yang et al., 2018; Bidkar et al., 2019; Huang et al., 2019; Lee et al., 2019; Wang et al., 2020b; Cao et al., 2020; Wang et al., 2020c; Li et al., 2020; Peng et al., 2020; Poudel et al., 2020; Wu et al., 2020; Chen et al., 2021a; Li et al., 2021a; Wang et al., 2021a; Zhao et al., 2021a; Wu et al., 2021b; Fan et al., 2021; Liu et al., 2021; Shen et al., 2021; Zhang et al., 2021). The pellet of cell membrane vesicles obtained is suspended in adequate phosphate-buffered saline and lyophilized for subsequent use. Microfluidic electroporation is another approach to make cell plasm membrane as shell (Molinaro et al., 2018; Castro et al., 2021). Different synthetic cores and source cells for membrane camouflaged NPs are summarized in Table 2.

**TABLE 2:** Different synthetic cores and source cells for membrane camouflaged nanoparticles and potential application.

Core material	Therapeutic agents	Source cell	Methods for coating	Potential utility	References
PLGA NPs	-	RBC	Extrusion	Drug loading	(Hu et al., 2011) Hu et al. (2011)
MNCs	-	RBC	Sonication	PTT	(Wang et al., 2020a) Wang et al. (2020)
Au NRs; TiO2 NPs	-	RBC	Sonication and extrusion	PTT	(Li and Zhang, 2020) Li et al. (2020)
Polypyrrole NPs	-	Erythrocyte-platelet	Extrusion	PTT	(Liu et al., 2018) Liu et al. (2018)
^89^Zr-HMSNs	-	RBC	Extrusion	Tumor diagnosis and treatment	(Lee et al., 2019) Lee et al. (2019)
Lipids	-	RBC	Sonication and extrusion	PTT	(Wu et al., 2021b) Wu et al. (2021)
BPQDs	DOX; Kirenol	RBC	Sonication; extrusion	Chemotherapy; anti-inflammatory therapy	(Huang et al., 2019) Huang et al. (2019)
MMSNs	-	RBC	Sonication	PDT	(Xuan et al., 2018) Xuan et al. (2018)
MSNs	-	RBC	Sonication	PTT	(Peng et al., 2020) Peng et al. (2020)
PAAO-UCNPs	Glucose oxidase	Cancer cell	Sonication	Starvation therapy; phototherapy	(Wang et al., 2020b) Wang et al. (2020)
PLGA NPs	Curcumin; Tirapazamine	RBC	Extrusion	Chemotherapy	(Bidkar et al., 2019) Bidkar et al. (2019)
PLGA NPs	DOX	RBC	Sonication	PTT; Chemotherapy	(Wang et al., 2020c) Wang et al. (2020)
NanoPorous Silicon particles	DOX	Macrophage; THP-1 phagocytic cell	-	Chemotherapy	(Parodi et al., 2013) Parodi et al. (2013)
PLGA NPs	-	Neutrophil	Extrusion	PDT	(Zhang et al., 2021) Zhang et al. (2021)
Liposomes	Ac4GalNAz/Ac4ManNAz	Cancer cell	Extrusion	Personalized diagnosis and treatment	(Liu et al., 2021) Liu et al
					(2021)
Liposomes	DOX	NK cell	Extrusion	Chemotherapy	(Pitchaimani et al., 2018) Pitchaimani et al. (2018)
PLGA NPs	PTX	Neutrophil	Sonication; extrusion	Chemotherapy	(Wang et al., 2021a) Wang et al. (2021)
Albumin NPs	PTX	Macrophage	Extrusion	Chemotherapy	(Cao et al., 2020) Cao et al (2020)
CuSNPs	PTX	Macrophage	Extrusion	PTT; PDT; Chemotherapy	(Poudel et al., 2020) Poudel et al. (2020)
Gelatin nanogels	DOX	Stem cell	Extrusion	Chemotherapy	(Gao et al., 2016) Gao et al. (2016)
PLGA NPs	DOX	Stem cell	Sonication	Chemotherapy	(Yang et al., 2018)Yang et al. (2018)
PLGA NPs	PTX	T-lymphocyte	Extrusion	Chemotherapy; radiotherapy	(Zhang et al., 2017) Zhang et al. (2017)
Polypyrrole NPs	DOX	Platelet	Extrusion	PTT; Chemotherapy	(Wu et al., 2020) Wu et al. (2020)
MSNs	Combretastatin A4; Apatinib	Platelet	Sonication	Chemotherapy	(Li et al., 2021a) Li et al. (2021)
PFCE-PLGA NPs	-	Cancer cell	Extrusion	PTT; tri-modal imaging	(Li et al., 2020) Li et al. (2020)
Catalase-HMSN	-	Cancer cell	Sonication; extrusion	PTT; PDT	(Chen et al., 2021a) Chen et al. (2021)
MSNs	ISOIM	Cancer cell	Sonication; extrusion	Chemotherapy	(Zhao et al., 2021a) Zhao et al. (2021)
Ir-B-TiO2 NPs	-	Cancer cell	Extrusion	PTT; SDT	(Shen et al., 2021) Shen et al. (2021)
HCPT-NS	-	Cancer cell	Sonication	Chemotherapy	(Fan et al., 2021) Fan et al. (2021)
PLGA NPs	β-mangostin	Platelet-Tumor cell	Extrusion	Chemotherapy	(Wu et al., 2021c) Wu et al. (2021)
PLGA NPs	DOX	Macrophage-cancer cell	Sonication	Chemotherapy	(Gong et al., 2020) Gong et al. (2020)
Liposomes	Proteins	Stem cell; neuron	Microfluidic	Neuron targeting	(Zinger et al., 2021) Zinger et al. (2021)
MSNs	-	T-lymphocyte	Sonication; extrusion	PTT	(Ma et al., 2020) Ma et al. (2020)
PLGA NPs	-	Cancer cell	Sonication	Immunotherapy	(Jiang et al., 2020a) Jiang et al. (2020)
MSV	-	Macrophage; T lymphocyte	-	-	(Evangelopoulos et al., 2016) Evangelopoulos et al. (2016)
PDA NPs	DOX; PD-L1 siRNA	Stem cell	Extrusion	Chemotherapy; Immunotherapy	(Mu et al., 2021) Mu et al. (2021)
Chitosan NPs	TNFα	Macrophage	Extrusion	Immunotherapy	(Bhattacharyya and Ghosh, 2020) Bhattacharyya et al. (2020)
Fe_3_O_4_ NPs	-	Erythrocyte-cancer cell	Sonication	PTT; immunotherapy	(Xiong et al., 2021) Xiong et al. (2021)
BMSNRs	-	Platelet	Sonication	PTT; radiotherapy	(Chen et al., 2019) Chen et al. (2019)
Liposomes	-	Macrophage; T-lymphocyte	Microfluidic	-	(Molinaro et al., 2018) Molinaro et al. (2018)
CS-pPLGA NPs	Bufalin	Platelet	Sonication; extrusion	Chemotherapy	(Wang et al., 2019) Wang et al. (2019)
PLGA NPs	PTX	143B epithelioid cell-RAW264.7 cell	Sonication; extrusion	Chemotherapy	(Cai et al., 2022) Cai et al. (2022)
MSN	DOX	Stem cell	Sonication	Chemotherapy	(Li et al., 2021b) Li et al. (2021)
Fe_3_O_4_ NPs	Sulfasalazine	Platelet	Extrusion	Immunotherapy	(Jiang et al., 2020b) Jiang et al. (2020)
UCNPs; AuNPs	-	Cancer cell	Extrusion	PTT	(Wang et al., 2020d) Wang et al. (2020)
UCNPs	-	Macrophage	Extrusion	PDT; immunotherapy	(Chen et al., 2021b) Chen et al. (2021)
UCNPs	PTD	Cancer cell	Extrusion	PDT; chemotherapy	(Jin et al., 2021) Jin et al. (2021)
CANS	-	Platelet	Extrusion	Brachytherapy	(Lyu et al., 2021) Lyu et al. (2021)
MSNs	DOX; SM	Neutrophil	Sonication; extrusion	Chemotherapy; anti-inflammatory therapy	(Zhao et al., 2021b) Zhao et al. (2021)
Nanoporous silicon particles	-	T-lymphocyte; Macrophage	-	-	(Palomba et al., 2016) Palomba et al. (2016)
MUNs	DOX	Cancer cell	Extrusion	PTT; PDT; Chemotherapy	(Xu et al., 2021) Xu et al. (2021)
ICG/DOX nanocomplexes	DOX	RBC	Extrusion	PTT; PDT; Chemotherapy	(Wan et al., 2018) Wan et al. (2018)
Melanin NPs	-	Erythrocyte-cancer cell	Sonication; extrusion	PTT	(Jiang et al., 2019) Jiang et al. (2019)
PNs	-	RBC	Sonication	Chemotherapy	(Zhai et al., 2020) Zhai et al. (2020)
PEG-b-PDLLA	PTX dimer	RBC	Extrusion	PDT; Chemotherapy	(Pei et al., 2018) Pei et al. (2018)
PLGA NPs	-	Platelet	Sonication	PDT	(Xu et al., 2018) Xu et al. (2018)
miR155-nanogel	miR155	RBC	Extrusion	Immunotherapy	(Gao et al., 2021) Gao et al. (2021)
CuS NPs	Sorafenib; Anti-VEGFR antibody	Macrophage-cancer cell	Sonication	PTT; chemotherapy	(Ji et al., 2020) Ji et al. (2020)

BPQDs: black phosphorus nanoparticle quantum dots; DOX: doxorubicin; EM: enaminitrile molecule; HCPT:10-hydroxycamptothecin; hollow mesoporous silica nanospheres; ICG: indocyanine green; HMSNs: MMSNs: Magnetic mesoporous silica nanoparticles; MSNs: mesoporous silica nanoparticles; MSV: multistage nanovector; NK: natural killer; NIR: near-infrared; NP: nanoparticle; NS: nanosuspension PDT: photodynamic therapy; PFCE: Perflfluoro-15-crown-5-ether; PAAO: polyacrylic acid-n -octylamine; PNs: PTX nanoparticles; PTD: polyethylene glycol-thioketal-doxorubicin; PTX: Paclitaxel; PTT: photothermal therapy; PLGA: poly (Lactic-co-glycolic acid); RBC: red blood cell; RT: brachytherapy; SDT: sonodynamic therapy; SM: shanzhiside methylester; TNFα: tumor necrosis factor-α; UCNP: upconversion nanoparticle; VEGFR: vascular endothelial growth factor receptor.

### 2.1 Cores of cell membrane-camouflaged NPs

In living beings, the core part of life is covered by a coating structure, along with the cell and some organelles. A functional artificial entity may be fabricated as a complex biomimetic entity consisting of a core and outer shell, with versatile functions that interact and fit with the environment and circumstances, thus enabling it to perform its core function (Attia et al., 2019; Dash et al., 2020; Wu et al., 2021a; Castro et al., 2021; Jha et al., 2021). The core constituents of cell membrane-camouflaged NPs can be organic, inorganic, or organic–inorganic hybrid nanosystems (Attia et al., 2019; Dash et al., 2020; Wu et al., 2021a; Castro et al., 2021; Jha et al., 2021). Typically, the core of the NP, which must be delivered and released at the target site, consists of its therapeutic agent and framework, which are shielded by cell membranes isolated from source cells (Gao et al., 2016; Zhang et al., 2017; Pitchaimani et al., 2018; Yang et al., 2018; Cao et al., 2020; Gong et al., 2020; Li et al., 2020; Poudel et al., 2020; Wu et al., 2020; Chen et al., 2021a; Li et al., 2021a; Wang et al., 2021a; Zhao et al., 2021a; Wu et al., 2021c; Fan et al., 2021; Shen et al., 2021).

#### 2.1.1 Organic NPs

Organic NPs are fabricated using organic compounds, such as polymers, liposomes, gelatin, chitosan, and human serum albumin (Lai et al., 2014; Gao et al., 2016; Liu et al., 2018; Pitchaimani et al., 2018; Bidkar et al., 2019; Wang et al., 2019; Cao et al., 2020; Poudel et al., 2020; Wu et al., 2020; Wang et al., 2021a; Liu et al., 2021; Ma et al., 2021; Zhang et al., 2021; Cai et al., 2022). Poly (lactic-co-glycolic acid) (PLGA) is an organic biodegradable and biocompatible compound and can be used for preparing polymeric core NPs as the skeleton of the core (Lai et al., 2014; Bidkar et al., 2019; Wang et al., 2020c; Zhang et al., 2021). These polymeric NPs, when loaded with anticancer drugs, near-infrared (NIR) dye, and/or other materials, can be used in comprehensive antitumor therapy or a combination of imaging and therapy (Lai et al., 2014; Bidkar et al., 2019; Wang et al., 2020c; Zhang et al., 2021).

Using PLGA to prepare drug loading NPs has been popular in recent decades. Bidkar et al. (Bidkar et al., 2019) developed RBC membrane-coated PLGA NPs for the co-delivery of chemotherapeutic drugs. In their work, curcumin (Cur), and the hypoxia-activated molecule tirapazamine (TPZ) (Cur + TPZ@RB) by an extrusion process. Compared with free drugs, Cur + TPZ@RB NPs had a stronger antiproliferative effect by generating reactive oxygen species (ROS) and thus DNA damage, thereby including apoptosis; accordingly, reduced cell migration and downregulations of mesenchymal markers were observed after Cur + TPZ@RB NP treatment. At 105 nm in size, the NPs were smaller than RBCs. The NPs exhibited prolonged circulation stability, superb biocompatibility, and efficient cellular internalization, and they could diffuse out of capillaries and into solid tumors, thereby significantly increasing their numbers in tumors, reducing hypoxic conditions, and supporting cancer therapy.

Zhang et al. (Zhang et al., 2021), meanwhile, developed neutrophil membrane-camouflaged PLGA NPs (NM-HB NPs) for synchronous near-infrared fluorescence (NIR FL) imaging and photodynamic therapy (PDT) against hepatocellular carcinoma using a broad-spectrum anti-inflammatory strategy. The NM-HB NPs were targeted effectively to the tumor site and could overcome the drawbacks of a short blood circulation time and high immune clearance *in vivo* and *in vitro*, and they showed significant PDT efficacy and suppression of tumor growth.

Polypyrrole (PPy) is a conductive polymer, the chemical structure comprises repeating units of the py monomer, a nitrogen-containing aromatic ring. It can be conjugated with biomolecules by chemical modification. PPy has high stability and superior biocompatibility. In tissue engineering, it can be used to fabricate biocompatible stimulus-responsive scaffolds. PPy can efficiently convert absorbed NIR light into heat, and can be used as photothermal therapy (PTT) agent for cancer therapy (Liu et al., 2018; Wu et al., 2020). Wu et al. (Wu et al., 2020) constructed PLT-PPy–DOX NPs for PTT and chemotherapy of hepatocellular carcinoma. The core NPs were PPy loaded with DOX, and membrane vesicles isolated from platelet were coated onto core NPs as shell. Further study showed that these NPs had good immune evasiveness and tumor targeting abilities *in vivo*; when under 808 nm laser irradiation, PPy in the NPs generated high heat to ablate tumors while DOX was released to kill cancer cells, which effectively suppressed the growth of primary tumor and inhibited tumor metastases.

Chitosan

Chitosan is a polycationic macromolecules derived from the alkaline deacetylation of chitin, and it is composed of randomly distributed β-(1-4)-linked d-glucosamine and *N*-acetyl-d-glucosamine (Bhattacharyya and Ghosh, 2020; Ma et al., 2021). It is a porous material with a high surface areas and interconnected pore channels, and it is a good candidate to be used as an adsorbent. In that vein, Ma et al. (Ma et al., 2021) employed a one-pot method to fabricate gold-embedded chitosan NPs (Au@CS NPs), which were modified by applying benzaldehyde-terminated poly [(2-methacryloyloxy) ethyl phosphorylcholine] (PMPC). The obtained Au@CS-PMPC NPs had a diameter of 135 nm and showed features of elevated colloidal stability, high loading capacity of drugs, pH-responsive drug release, superior biocompatibility, and marked fluorescence emission. Bhattacharyya et al. (Bhattacharyya and Ghosh, 2020), meanwhile, developed TNFα-expressed macrophage membrane-coated chitosan NPs. The chitosan core NPs were synthesized using the ionic gelation method, and macrophage membranes were coated on the core NPs by serially extruding the membranes through 0.8 and 0.4 μm-pore membranes, followed by coextrusion of the cell membranes and NPs, with a 0.2 μm-pore membrane in the extruder.

Gelatin

Gelatin is another type of organic material used for cell membranes cloaked NPs, and it is a mixture of peptides and proteins. Gao et al. fabricated bone marrow-derived stem cell membranes camouflaged gelatin nanogels by preparing gelatin nanogels, loading with DOX, and then coating them with mesenchymal stem cells (MSCs) membranes (SCMGs-DOX) using a top-down protocol (Gao et al., 2016). The SCMGs-DOX had the innate functions of natural MSCs membranes and demonstrated a strong ability to evade immune system clearance, along with having an improved tumor-targeting capability and enhanced antitumor efficacy.

Liposomes

Liposomes possess hydrophilic and hydrophobic properties that can be used as the core material for loading both hydrophilic and hydrophobic therapeutic cargos (Pitchaimani et al., 2018; Liu et al., 2021). Liu et al. (Liu et al., 2021) constructed cancer cell membrane–camouflaged azido sugar liposomes with a feature of cell-selective glycan imaging. In this work, the core NPs were azido sugar liposomes, and cell membranes of cervical and breast cancer were coated onto the core NPs through sonication and extrusion. These liposomes can image different cancer cells and subtype cells of triple-negative breast cancer, as well as to label metastatic tumors, with features of pH-responsive, preventing protein corona formation, immune evasion, extension of blood circulation time, facilitating metabolic glycan labeling, and increased prominent cell selectivity to homotypic cancer cells. In another study, Pitchaimani et al. (Pitchaimani et al., 2018) also successfully developed biomimetic NPs with liposomes for anticancer. They developed DOX-loaded biomimetic liposomes using thin-film hydration and membrane extrusion techniques, with NK cell membranes for camouflaging.

#### 2.1.2 Inorganic NPs

Inorganic NPs are synthetic nanoscale materials that are used as cores, and which can be cloaked with different cellular membranes for various aims of application. Inorganic NPs with tunable and diverse properties hold great potential in the field of nanomedicine, but their non-negligible toxicity for healthy tissues and organs has restricted their clinical use (Wang et al., 2021b). There are many inorganic materials available for biomedical applications, and the most commonly used inorganics are silicon, Fe_3_O_4_ and upconverted materials.

Silica

Compared with other porous silica nanocarriers, mesoporous silica NPs, with a pore size ranging from 2 to 50 nm, high capability of therapeutic cargo loading and a highly biocompatible nature, make excellent candidates for drug delivery and biomedical applications (Wu et al., 2021a; Wang et al., 2021b). Accordingly, biomedical silica NPs are developed for cargo delivery, disease treatment, and bioimaging. To develop imaging-guided tumor PTT, Peng et al. (Peng et al., 2020) prepared MSN-ICG@RBC NPs by forming mesoporous silica NPs (MSNs), modifying their surface functionalization in various ways (-COOH, -SH, -NH_2_), conjugating them with ICG, and coating RBC membrane ghosts. The MSN-ICG@RBC NPs with an optimized particle size of 60 nm showed prolonged blood circulation, higher accumulation at target sites, and efficient imaging-guided PTT. Li et al. (Li et al., 2021b), meanwhile, developed a DOX-loaded MSN@M for antitumor treatment. The membranes of MSCs were coated onto DOX-loaded mesoporous silica NPs (MSN@M). Subsequent study showed the NPs had good self-positioning drug delivery ability, immune evasion, stronger tumor targeting and penetration, effective tumor inhibition, and minimal side effects.

Magnetic NPs

Magnetic NPs can be magnetized under an external magnetic field; accordingly, they can be used for alternate magnetic field-mediated hyperthermia and magnetic resonance imaging. In a study by Wang et al. (Wang et al., 2020a), biomimetic Cyp-MNC@RBCs were established for bimodal NIR fluorescence and magnetic resonance imaging-guided cancer PTT. The superparamagnetic nanoclusters (MNCs) had an average diameter of 90 nm and were fabricated using iron (iii) chloride hexahydrate (FeCl_3_.6H_2_O) as the precursor by a modified solvothermal reaction method. A NIR with a peak at 785 nm, containing carboxyl groups with cypate, was loaded into the MNCs through coordinated chemical interactions between the Fe atoms and carboxyl groups and, then, RBC membranes were coated on MNCs to form Cyp-MNC@RBCs. The Cyp-MNC@RBCs demonstrated excellent biocompatibility, prolonged blood circulation, superior tumor-homing capacity, and improved photothermal conversion ability when compared with their MNCs counterparts. Treated with a Cyp-MNC@RBC injection and 808 nm laser irradiation, the growth of tumors was effectively suppressed.

In further work, Wang et al. (Wang et al., 2020c) fabricated magnetically targeted RBC membrane-camouflaged NPs of DOX@IRP@RBC for PTT and chemotherapy of cancers. DOX, IR-780 iodide, and Fe_3_O_4_ was loaded into the PLGA core NPs, then coated with RBC membranes by sonication. The DOX@IRP@RBC NPs exhibited good immune evasion, a prolonged blood circulation, good biocompatibility, high tumor accumulation, minimal systemic side effects; more so, an external magnetic field can be used for magnetic-guided targeted drug delivery and therapy.

Beyond this, to combat ovarian cancer, Xiong et al. (Xiong et al., 2021) fabricated biomimetic Fe_3_O_4_-ICG@IRM NPs for synergistic PTT and immunotherapy. ICG was loaded into magnetic NPs (Fe_3_O_4_) and then camouflaged with murine-derived ID8 ovarian cancer cell membranes and RBC membranes (IRM). The resulting Fe_3_O_4_-ICG@IRM NPs demonstrated highly specific self-recognition of ID8 cells*,* had a prolonged blood circulation, activated specific immunity in a tumor-bearing model, and supported synergistic PTT and antitumor immunotherapy for primary ovarian cancer and metastatic tumors.

NPs with Fe_3_O_4_ can trigger ferroptotic cell death, induce a tumor-specific immune response, and promote macrophages polarizing to an antitumor M1 phenotype, as reported by Jiang et al. (Jiang et al., 2020b). In their work, sulfasalazine (SAS) was loaded into the mesoporous magnetic NPs (Fe_3_O_4_), and then coated with platelet (PLT) membranes via extrusion. The prepared Fe_3_O_4_-SAS@PLT NPs exhibited high efficacy of cancer immunotherapy.

Upconverted NPs

Upconverted NPs are a group of inorganic fluorophores that utilize the anti-Stokes mechanism and can convert NIR wavelengths to visible or ultraviolet wavelengths under the assistance of dopant activators or emitter elements in the nanocrystal core (Corbo et al., 2016; Evangelopoulos et al., 2016; Bhattacharyya and Ghosh, 2020; Wang et al., 2020b; Ma et al., 2021). These NPs have some strengths, such as narrow emission peaks, low toxicity, exceptional photostability, a remarkable light-penetration depth, and negligible background fluorescence (Wang et al., 2020d; Chen et al., 2021b; Jin et al., 2021; Rostami, 2021). They can be used in tumor imaging or imaging-guided therapy. In a study, Wang et al. (Wang et al., 2020b) made use of upconverted materials to fabricate cancer cell membrane-coated NaYF_4_: Yb, Tm UCNPs for homotypic targeting cancer multimodal therapy, and which exhibited good performance *in vitro* and vivo.

In further work, Wang et al. (Wang et al., 2020d) fabricated cancer cell membrane-camouflaged UCNP/AuNPs for multimodal imaging-guided NIR PTT. The researchers produced cancer cell membrane-cloaked upconverted NPs (CC-UCNPs) and gold NPs (CC-AuNPs). These NPs allowed simultaneous dual-modal imaging on a dual-modal imaging system with a special designed detector that can simultaneously detect both high-energy X-ray and low-energy visible light. Further *in vitro* and *in vivo* studies showed these NPs had highly specific upconverted luminescence imaging and PTT-based anti-tumor efficacy, with abilities of superior immune evasion, a long blood circulation time, and high tumor-targeting specificity. Beyond this, Chen et al. (Chen et al., 2021b) developed NPR@TAMM NPs by synthesizing NaYF4:Yb, Er upconverted NPs, which were then loaded with PS, to yield NPR as the core NP, and coated with tumor-associated macrophage membranes (TAMM) as the shell. Further study showed that these NPs had good tumor-homing ability and immune evasion, and exhibited high performance of PDT and immunotherapy in antitumor.

### 2.2 Source cells used for camouflaging, membrane properties and their mechanisms

The source cells used for camouflaging core NPs mainly include RBCs, platelets, macrophages, NK cells, T lymphocytes (TLs), neutrophils, stem cells, and cancer cells, as listed in Table 1.

RBCs are the essential blood cells, with an exclusive function of carrying oxygen to the tissues and cells of the body. On RBCs membranes present surface proteins, such as CD47 that can bind receptors on the leukocyte membrane (i.e., SIRPα), inhibiting their clearance; and other “self-markers” on their surfaces that prevent clean by the reticuloendothelial system and prevent immune attack (Hu et al., 2011; Liu et al., 2018; Xuan et al., 2018; Bidkar et al., 2019; Huang et al., 2019; Lee et al., 2019; Wang et al., 2020a; Wang et al., 2020b; Li and Zhang, 2020; Peng et al., 2020; Wu et al., 2021b). Combined with the above properties, NPs coated with RBC membranes provide much longer plasmatic half-life, which making them suitable for use as long-circulating carriers.

Platelets contain unique surface moieties and have innate functions of binding injured blood vasculature, facilitating subendothelial adhesion, working with the immune system, and adhering to and interacting with pathogens (Chen et al., 2019; Wang et al., 2019; Jiang et al., 2020b; Wu et al., 2020; Li et al., 2021a; Lyu et al., 2021) Platelet membrane-camouflaged NPs can escape immune attack and bind to injured blood vessels and some pathogens, which allows the core NPs to deliver their payload.

Neutrophils are granulocytes in the white blood cells that abundantly present in the blood. Neutrophils are not confined in the blood circulation and can move through capillary walls to tissues to instantly attack antigens (Jaillon et al., 2020; Wang et al., 2021a; Zhao et al., 2021b; Zhang et al., 2021). Neutrophils are a key component of the body immune system and are functioned in protecting the body from contagious diseases and foreign invaders; they also play a crucial role in tumor progression. Tumors recruit neutrophils by secreting a chemoattractant (Jaillon et al., 2020). Neutrophile membranes have properties of immune evasion and target tissue localization, the latter of which functions via cell–cell interactions (Wang et al., 2021a; Zhao et al., 2021b; Zhang et al., 2021). As for the former, NPs camouflaged with neutrophil membranes can escape immune system processes of opsonization and uptake (Wang et al., 2021a; Zhao et al., 2021b; Zhang et al., 2021).

Macrophages develop from monocytes in the bone marrow and many forms of mononuclear phagocytes can be found in tissues; and these phagocytes can replenish themselves in peripheral tissues directly from local precursors (Xia et al., 2020). Macrophages have functions in identifying, intaking, and digesting cellular debris and other foreign substances (Cao et al., 2020; Poudel et al., 2020; Xia et al., 2020). In addition, macrophages can present antigens to T cells and release cytokines that initiate inflammation and activate other cells. Macrophages in the tumor microenvironment are often associated with tumor progression and metastasis (Parodi et al., 2013; Evangelopoulos et al., 2016; Molinaro et al., 2018; Bhattacharyya and Ghosh, 2020; Cao et al., 2020; Gong et al., 2020; Poudel et al., 2020; Chen et al., 2021b). Macrophage membrane-camouflaged NPs show excellent biocompatibility, prolonged blood circulation, selective tumor site accumulation, and strong cellular internalization and antitumor efficacy (Parodi et al., 2013; Evangelopoulos et al., 2016; Molinaro et al., 2018; Bhattacharyya and Ghosh, 2020; Cao et al., 2020; Gong et al., 2020; Poudel et al., 2020; Chen et al., 2021b).

A previous study indicated that NPs camouflaged with purified leukocyte (human T-cell and murine macrophage) membranes presented cell-like behaviour in the blood circulation in the orthotopic 4T1 tumor models, which could molecularly interact with the surface of the cell through triggering and activating the clustering of intercellular adhesion molecule one on endothelial cells, increasing the targeting properties, promoting firm adhesion to the tumor vasculature, resulting in an increase in intracellular calcium and ROS concentrations, resulting in an independent activation of PKCα. PKCα increases lead to the phosphorylation of vascular endothelial-cadherin (VE-cadherin, also known as cadherin-5 and CD144) that resulting in the disassembly of VE-cadherin and protein displacement, leading to enlarged gaps between endothelial cells and an increase in tumor vascular permeability (Palomba et al., 2016).

NK cells are lymphocytes in the same family as T and B cells, which all originate from a common progenitor (Pitchaimani et al., 2018). NK cells belong to group I innate lymphocytes and respond quickly to many pathological challenges, such as killing cells infected by virus and detecting and controlling cancer in early stage. NK cells can attack cancer cells directly through inhibitory and activating receptors on their cell surfaces and can also cause cell killing without prior sensitization (in contrast to cytotoxic T cells, which require priming by antigen-presenting cells) (Pitchaimani et al., 2018). Coated with NK cell membranes, a biomimetic system of DOX-loaded NPs exhibited high affinity toward cancer, excellent tumor-homing efficacy and antitumor activity, while these depended exclusively upon the membrane features of NK-92 cell membrane receptors (Pitchaimani et al., 2018).

Cytotoxic T lymphocytes (CTLs) are another type of immune cells. They can kill cancer cells and other infected cells (Evangelopoulos et al., 2016; Palomba et al., 2016; Zhang et al., 2017; Ma et al., 2020), and facilitate chosen target cell death via granule and receptor-mediated mechanisms. CTL-mediated immunity relies on granzymes, and gasdermin-mediated pyroptosis has been identified as a CTL-killing mechanism (Zhou et al., 2020). CTLs have innate properties that recognize T cell receptors on target cells and antigen-derived peptide fragments on the cells’ surfaces with specificity for antigens, which locate in the grooves of class 1 MHC (Raskov et al., 2021). Except for as mediators of antitumor immunity, CTLs are good candidates for the treatment of some systemic diseases, for CTLs can circulate throughout the body repeatedly and can find out antigens. One of the mechanisms of MHC class 1 in immune response is to activate cytolysis by recognizing a single peptide. To the end, CTLs may work by employing non-effector mechanisms along with the production of a cytokine of interferon-gamma composing of several antitumor properties (Zhou et al., 2020; Raskov et al., 2021). CTL membrane-camouflaged cargo-loaded NPs can avoid opsonization thanks to their prolonged circulation time and can localize and accumulate at the tumor site, thus bearing enhanced targeting ability (Zhang et al., 2017).

Cancer cells have a natural property that one adheres to one another, thus allowing tumor to continuous growing, known as homotypic binding. A study indicated that interactions of surface adhesion molecules like galectin-3 and Thomsen-Friedenreich antigen (T antigen) are involved in carbohydrate-mediated metastatic cell heterotypic (between carcinoma cells and endothelium) and homotypic (between carcinoma cells) adhesion (Zou et al., 2005); and similar phenomena was found in another study with different cancer cells (Khaldoyanidi et al., 2003). Making use of the homotypic binding property of membranes of different cancer cells during the contact of the tumor cell membrane, individualized cancer cell membrane-coated biomimetic NPs can be developed to aid homotypic targeting and facilitate internalization by source cells thanks to their self-recognition properties (Li et al., 2020; Chen et al., 2021a; Zhao et al., 2021a; Fan et al., 2021; Shen et al., 2021). Homotypic cell membranes increase the chances of NP-to-cell adhesion and may target different sites with cancer metastasis potential. Cancer cell membranes coated NPs have similar cell adhesion molecules to those of their source cells. The ability of cancer cell membranes coated NPs to initiate homologous targeting with innate self-adhesive properties allows for augmented delivery of different anticancer payloads to the tumor. The preferential accumulation of cancer cell membrane NPs in the tumor improves the therapeutic efficacy as well as reduces the systemic toxicity. These made the cancer cell membranes coated NPs have a variety of applications, including drug delivery, and image-guided PTT and sonodynamic therapy (SDT), anticancer vaccination, targeted oxygen interference for chemoresistance to increase the therapeutic effect (Li et al., 2020; Chen et al., 2021a; Zhao et al., 2021a; Fan et al., 2021; Jin et al., 2021; Liu et al., 2021; Shen et al., 2021; Xu et al., 2021). NPs coated with cancer cell membranes can effectively deliver tumor-associated membrane-bound antigens with immunological adjuvants to antigen-presenting cells by inducing the anticancer immune response (Li et al., 2020; Chen et al., 2021a; Zhao et al., 2021a; Fan et al., 2021; Jin et al., 2021; Shen et al., 2021).

Stem cells can self-renew with high replicative potential in multilineage differentiation (Gao et al., 2016; Yang et al., 2018; Li et al., 2021b; Mu et al., 2021; Zinger et al., 2021). Embryonic stem cells are generally used for therapeutic purposes because of their superb totipotency and long lifespan. MSCs have the unique ability to home and engraft in tumor stroma. MSCs homing is a process that endogenous or exogenous MSCs migrate to the targeted tissue and colonize under the action of relevant factors. Different signaling molecules generated from different microenvironments and attract MSCs to these sites. Bone marrow, various organs and even tumor are the eventual sites of MSCs homing. Studies showed that some ligands and corresponding receptors involve in MSCs migration, such as SDF-1/CXCR4, PDGF/PDGFR, and VEGF/VEGFR (Yang et al., 2018). These specific signaling molecules secreted by tumor cells can bind to corresponding proteins on the surface MSCs and result in the homing behavior of MSCs. The vascular endothelial growth factors formed at the tumors allowing the recruitment of MSCs for the formation of tumoral stroma and pericytes aimed at angiogenesis (Gao et al., 2016; Yang et al., 2018; Li et al., 2021b). It is believed that MSCs homing is similar to the chemotaxis of immune cells to the damaged or stimulated site. The exact mechanism of MSCs homing to tumor remains unclear (Gao et al., 2016; Yang et al., 2018; Mu et al., 2021). Because MSCs hardly express MHC molecules that may trigger immune response, so tumor targeting of MSCs has no species-specific property, this allows the alternative for clinical application of MSCs derived from other species (Wang et al., 2019; Zinger et al., 2021). These features render them potentially a very useful tool as MSC have been used for treatment in several fields, from the treatment of graft-versus-host-disease to tissue engineering and design of targeted delivery vehicles which can deliver antitumor therapeutic cargos to the tumor, with the aim of enhancing drugs selective accumulation at the tumor sites (Yang et al., 2018; Li et al., 2021b; Mu et al., 2021; Zinger et al., 2021).

In general, the membranes obtained from source cells have innate properties; NPs camouflaged with such cell membranes can avoid immune clearance and benefit from a prolonged systemic circulation time. Stem cell membrane-coated NPs can thus help target cancer, and cancer cell membrane-coated NPs exhibit homologous tumor-targeting because of their homotypic binding.

## 3 Process of fabricating cell membrane camouflaged NPs

Generally, cell membrane-camouflaged NPs are fabricated in three steps: the preparation of core NPs, isolation of cell membranes and formation of membrane vesicles, and fusion of core NPs with those vesicles by extrusion, sonication, a combination of sonication and extrusion, or microfluidic electroporation (Parodi et al., 2013; Gao et al., 2016; Zhang et al., 2017; Pei et al., 2018; Pitchaimani et al., 2018; Wan et al., 2018; Xu et al., 2018; Xuan et al., 2018; Yang et al., 2018; Bidkar et al., 2019; Huang et al., 2019; Jiang et al., 2019; Lee et al., 2019; Li et al., 2019; Wang et al., 2019; Wang et al., 2020b; Cao et al., 2020; Wang et al., 2020c; Gong et al., 2020; Li et al., 2020; Peng et al., 2020; Poudel et al., 2020; Wu et al., 2020; Zhai et al., 2020; Chen et al., 2021a; Li et al., 2021a; Wang et al., 2021a; Zhao et al., 2021a; Wu et al., 2021b; Wu et al., 2021c; Fan et al., 2021; Gao et al., 2021; Jha et al., 2021; Liu et al., 2021; Mu et al., 2021; Shen et al., 2021; Zhang et al., 2021), as listed in Table 2. In the process of extrusion, both the membrane vesicles and core NPs are coextruded many times through a porous membrane with the same or different pore size (polycarbonate, polyester) (Pitchaimani et al., 2018; Huang et al., 2019; Li and Zhang, 2020; Wu et al., 2021b). Ultrasound sonication is a procedure that putting the core NPs and membrane vesicles in the same container, using ultrasonic waves with a certain power to form membrane-coated NPs. A drawback of the sonication method is that the membrane-coating formed might be uniformity and the NPs may be polydisperse (Huang et al., 2019; Wang et al., 2020c; Jha et al., 2021; Liu et al., 2021). Electroporation, meanwhile, refers to the application of strong external electric-field pulses to cells and tissues to structurally rearrange cell membranes, causing multiple pores to form in these membranes (Balantič et al., 2021; Jha et al., 2021), through which the core NPs diffuse into cells. Though the purposes of transferring biological features from cell membranes to synthetic NPs for therapeutic cargos delivery have been achieved, a standardizable, batch-to-batch consistent, scalable, and high-throughput assembly method has not been well developed. Microfluidics may be a promising tool for the controlled synthesis of NPs in a versatile and reproducible approach (Molinaro et al., 2018; Zinger et al., 2021).

## 4 Fabrication and biomedical presentation of different cell membrane- camouflaged NPs

Coating core NPs with cell membranes reserves the physicochemical properties of NPs while adds the cellular membrane functions of the source cells. These source cell membrane-camouflaged NPs have improved biocompatibility, enhanced immune evasion, increased tumor-targeting ability, and implemented engineered membrane peptides and motifs transferring. As a result, deriving cellular membranes from source cells, to use for anticancer NP camouflaging, has become a common practice (Table 1).

### 4.1 Erythrocyte membrane-camouflaged NPs

Many studies have been conducted on NPs camouflaged with membranes derived from RBCs (Khaldoyanidi et al., 2003; Hu et al., 2011; Liu et al., 2018; Wan et al., 2018; Xuan et al., 2018; Bidkar et al., 2019; Huang et al., 2019; Jiang et al., 2019; Lee et al., 2019; Wang et al., 2020a; Wang et al., 2020c; Li and Zhang, 2020; Peng et al., 2020; Zhai et al., 2020; Wu et al., 2021b). RBCs were used in the first-reported membrane-camouflaged NPs (Hu et al., 2011), whereby Li et al. (Li and Zhang, 2020) constructed Au/TiO_2_@RBC NPs for tumor PDT by isolating RBC membranes and coating them on Au/TiO_2_ NPs via sonication and extrusion. Peng et al. (Peng et al., 2020) later enveloped ICG conjugated mesoporous silica NPs (MSNs) with RBC membranes using repeated extrusion to develop MSN-ICG@RBC NPs for cancer PTT, which demonstrated longer blood circulation and good target site accumulation.

**FIGURE 3:**
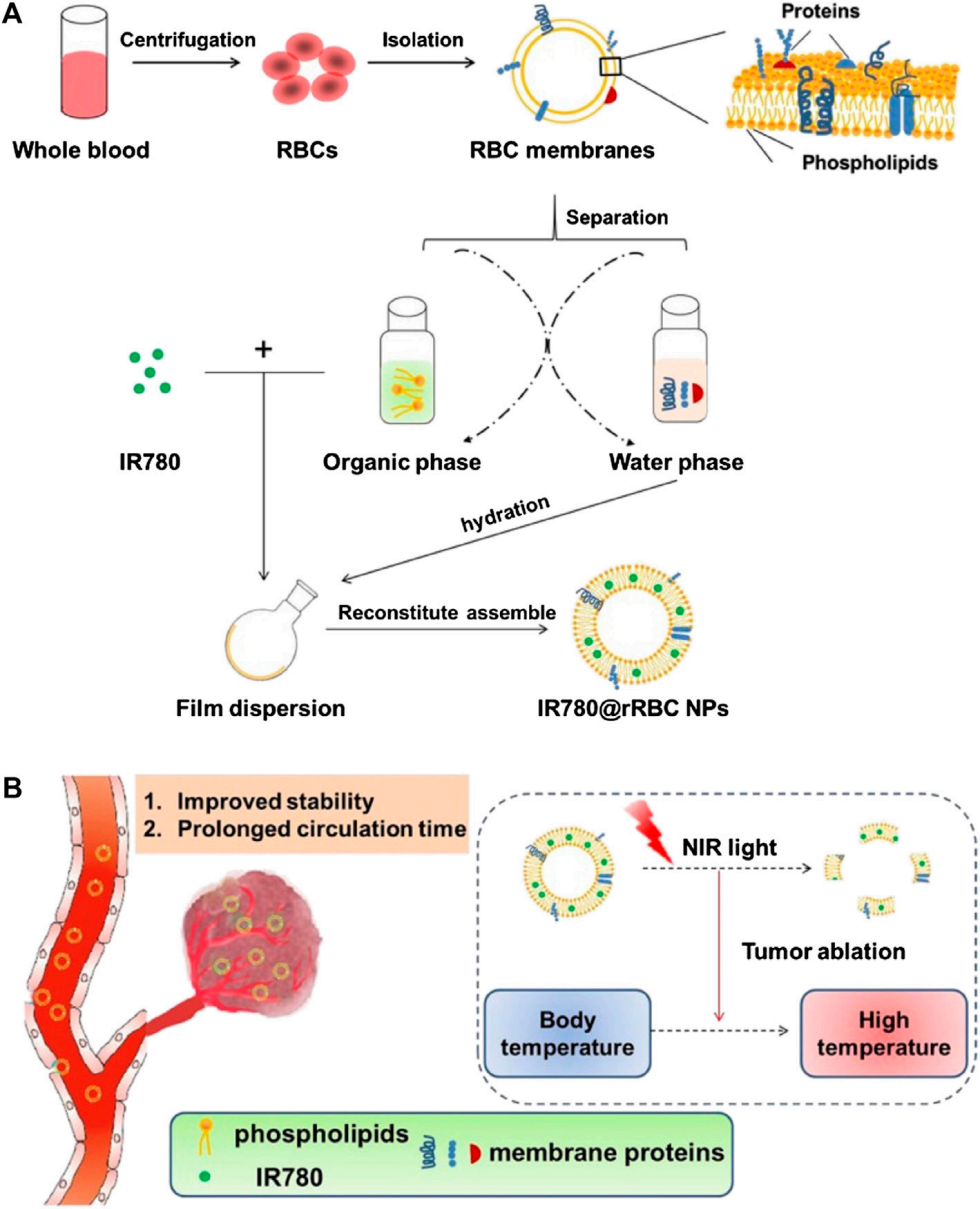
Schematic illustration of procedures for the preparation of IR780 loaded reconstitute RBC membrane nanoparticles (IR780@rRBC NPs) **(A)** RBC membrane was prepared by a hypotonic lysis method. Then RBC membranes were treated by mixed organic solvent, to separate the lipids and proteins. Lipid part was used to load IR780 by film dispersion method. At last, IR780@rRBC NPs were formed by adding proteins with the film, following further extrusion **(B)** IR780@rRBC NPs increased stability *in vitro*, and prolonged circulation capacity and enhanced PTT efficacy *in vivo*. IR780: IR780 iodide; PTT: photothermal therapy; RBC: red blood cell; rRBC: reconstitute RBC. Reproduced with permission from reference (Wu et al., 2021b). Copyrights ^©^ Springer Nature. 2022 BioMed Central.

Following on from this, Wu et al. (Wu et al., 2021b) formulated IR780@rRBC NPs for PTT anti-tumor, with recombinant RBC membranes as camouflage [Figure 3]. The RBC membranes were isolated using a hypotonic lysis method and then treated with a compound organic solvent to separate the lipids and proteins, after which the lipids were used to load IR780 iodide with the film dispersion method. IR780@rRBC NPs showed low toxicity, high stability, and optimized pharmacokinetics and pharmacodynamics.

In further work, RBC membrane-camouflaged NPs were also used for synergistic anticancer of PTT and chemotherapy (Khaldoyanidi et al., 2003; Jiang et al., 2019). Zhai et al. (Zhai et al., 2020) formulated a biomimetic PTX-NP-EM (PNM) system for anticancer therapy. Paclitaxel (PTX)-loaded polyethylene glycol (PEG) was used for the core NPs, and the erythrocyte membrane (EM) was isolated and utilized for cloaking via sonication and extrusion. The resulting system presented superior tumor cell uptake, stronger tumor cell killing, greater accumulation in tumors, and was markedly effective against tumor growth.

Nevertheless, RBC membrane coating of NPs should base on other important considerations in their design, including their shape or size, which influence significantly their fate *in vivo*. Such an example was seen in a work by Li et al. (Li et al., 2019) indicated that smaller, spherical RBCs membrane coated PLGA NPs (80 nm) had a longer blood serum half-life (30 h) and reduced liver accumulation compared to bigger NPs (100 and 200 nm, with an half-life around 10 h). The reason is that bigger NPs in liver filtration via sinusoid capillaries reduced. Thus, smaller particles appeared to be more suitable to enable long circulation time.

### 4.2 Platelet membrane-camouflaged NPs

Platelets are important for the maintenance of homeostasis. Platelet membranes contain the proteins, surface moieties and antigens present on the source platelets. Platelet membrane-camouflaged NPs have various biomedical applications, including drug delivery and anticancer therapy (Xu et al., 2018; Chen et al., 2019; Wang et al., 2019; Jiang et al., 2020b; Wu et al., 2020; Li et al., 2021a; Lyu et al., 2021). Platelet membranes can be used for camouflage with different aims of drug delivery and therapy. Li et al. (Li et al., 2021a) constructed MSN@PM-C-A NPs for antitumor treatment; mesoporous silica NPs (MSNs) loaded with CA4 antibodies and apatinib were the core NPs, and platelet membranes (PMs) were used as the shell. A further study in animal models showed that MSN@PM-C-A NPs accumulated in the tumor and targeted adhesion of the PM surface to the sites of damaged blood vessels in the tumor, resulting in significant vascular disruption and efficient anti-angiogenesis.

The similar merits of platelet membranes coating were exhibited in another study by Xu et al. (Xu et al., 2018). In their work, a biomimetic system of NP-Ver@P was developed for antitumor PDT. The core NPs were prepared using PLGA, with verteporfin NPs loaded, and sonication technique was used for platelet membranes camouflaging. Subsequent study showed that NP-Ver@P had an active targeting capacity, able to ablate tumors without causing skin damage. Platelet membranes camouflaging for NPs for synergistic of radiotherapy and PTT also indicated good performance. In another study by Chen et al. (Chen et al., 2019), BMSNR@PM NPs for tumor radiotherapy enhancement were formulated using solvothermal and ultrasonic methods. In their work, the core NPs were mesoporous silica-coated bismuth nanorods (BMSNR), and platelet membranes were used for coating. As a result, BMSNR@PM NPs showed a synergistic effect of radiotherapy *in vivo* with the photothermal effect induced by 808 nm NIR irradiation.

### 4.3 Neutrophile membrane-camouflaged NPs

Neutrophils play important roles in the protection of body from infection and in the activation and regulation of inherent and adaptive immunity. Neutrophils have properties of diversity and plasticity, these underlie the dual potential of tumor-associated neutrophils (TANs) in the tumor microenvironment (Jaillon et al., 2020). In the context of cancer, TANs have been an important component of the tumor microenvironment (Jaillon et al., 2020). TANs can contribute to tumor-promoting processes by promoting angiogenesis, extracellular matrix remodeling, metastasis, and immunosuppression. Conversely, neutrophils can also boost antitumor responses by directly killing tumor cells and incorporating into cellular networks that mediate antitumor resistance.

Using neutrophil membranes as NPs camouflaging were popular. Zhao et al. (Zhao et al., 2021b) developed Nm@MSNs-DOX/SM NPs for antitumor chemotherapy and anti-inflammatory therapy. They loaded mesoporous silica NPs (MSNs) with DOX, using the anti-inflammatory drug shanzhiside methylester (SM) for the preparation of core NPs and neutrophil membranes (Nm) as the shells. Subsequent study demonstrated that Nm@MSNs-DOX/SM NPs escaped the recognition of macrophages, reduced the phagocytosis of macrophages, exhibited excellent biocompatibility, increased the EPR effect and active tumor targeting, remodeled the tumor microenvironment, and amplified the antitumor effect of DOX. In another study by Zhang et al. (Zhang et al., 2021), the merits of neutrophil membranes camouflaging were validated too. In their work, PLGA NPs loaded with hypocrellin B were constructed as core NPs, and neutrophil membranes were used as the shell, with the aim for hepatocellular carcinoma PDT. Subsequent study showed that the fabricated NM-HB NPs adequately targeted the tumor and mitigated removal from the blood circulation and immune elimination.

### 4.4 Macrophage membrane-camouflaged NPs

A macrophage membrane-camouflaged NP contains associated membrane proteins of source macrophages, thus making it adequate for tumor targeting (Parodi et al., 2013; Evangelopoulos et al., 2016; Molinaro et al., 2018; Bhattacharyya and Ghosh, 2020; Cao et al., 2020; Gong et al., 2020; Poudel et al., 2020; Chen et al., 2021b). Macrophages have α-4 integrins on membranes, which have ability to actively bind to vascular adhesion molecule on cancer cells. In a study by Cao et al. (Cao et al., 2020), PTX-loaded RANPs were fabricated for targeted therapy against malignant melanoma. In their work, core NPs loaded with PTX were formulated via nanoprecipitation technique using human serum albumin; RAW 264.7 macrophage membranes and membrane vesicles were obtained using a combination of hypotonic lysis, mechanical membrane fragmentation, and differential centrifugation; and finally, the PTX-loaded RANPs were built via coextrusion of the membrane vesicles and the core NPs. A further study showed that the PTX-loaded RANPs had a prolonged blood circulation time, selective accumulation at the tumor, and markedly improved antitumor efficacy in tumor-bearing mouse xenografts.

In a further study by Bhattacharyya et al. (Bhattacharyya and Ghosh, 2020), a novel TNFα-expressed macrophage membrane was engineered as camouflaging shell for the fabrication of NPs for antitumor therapy. Transmembrane TNFα is a crucial signaling cytokine with anticell proliferative potential. In their work, bacterial lipopolysaccharide was used to induce the expression of membrane-bound TNFα through challenging phorbol 12-myristate 13-acetate-differentiated THP-1 cells, and the TNFα-expressed macrophage membranes were isolated and saved. Meanwhile, a well-established ionic gelation method was used to synthesize chitosan core NPs, and finally the core NPs were coated with the TNFα-expressed macrophage membranes through coextrusion. Subsequent study indicated that both the innate and acquired biological properties had been transferred to the fabricated NPs, which showed significant innate anti-cell proliferative potential, inherent capacity of immune escaping, excellent biocompatibility, and dose-dependent cell death in tumor spheroids.

### 4.5 Natural killing cell membrane-camouflaged NPs

NK cells are lymphocytes of the innate immune system that function between the innate and adaptive immunity. They play important roles for the defense against viral infections, tumor surveillance, autoimmune and inflammatory disorders, and atopic diseases (Pitchaimani et al., 2018; Dash et al., 2020; Jha et al., 2021). Biomimetic NPs camouflaged with NK membranes have high tumor-homing ability, excellent biocompatibility, can prevent immune evasion, as were proved in a study by Pitchaimani et al. (Pitchaimani et al., 2018). In their work, a biomimetic system of DOX@NKsome NPs was developed for targeted tumor therapy. In the process, cationic fusogenic liposome was prepared using thin-film hydration and extrusion techniques, anticancer drug DOX was loaded into the liposomes as the core NPs, and finally the isolated NK membranes were used for shell of camouflage.

### 4.6 T cell membrane-camouflaged NPs

Cytotoxic T cells have CD8 receptors that recognize antigens on the surfaces of virus-infected cells; once infected cells are detected, the cytotoxic T cells bind to and kill them. Cytotoxic T cell membrane-camouflaged NPs could thus be used as tumor‐targeting NPs.

Using T cell membranes as NPs camouflaging was not copious in the present literature, but from the available studies, its merits have presented. In a study by Zhang et al. (Zhang et al., 2017), human cytotoxic T lymphocyte membranes were used for fabricating TPNPs for the therapy of gastric cancer. In the process, the membrane vesicles were obtained by a combination techniques of hypotonic lysis, homogenizer disruption, centrifugation, and sonication, and finally the membrane vesicles were coated onto the core PTX-loaded PLGA NPs via coextrusion. Subsequent study showed that the TPNPs exhibited longer circulation time, good tumor site accumulation, and strong inhibition of the growth of human gastric cancer in mice after local low-dose irradiation.

**FIGURE 4:**
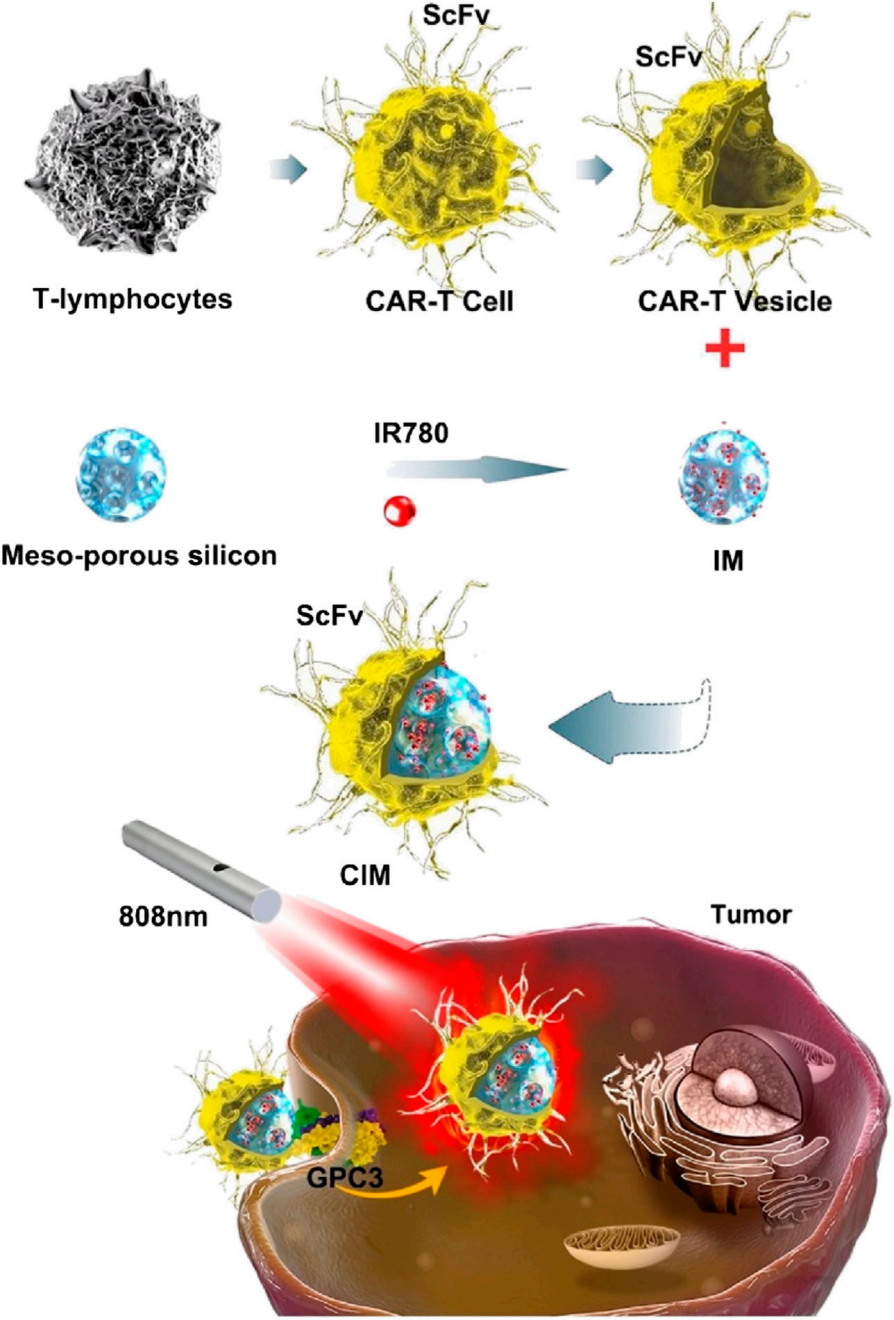
Schematic illustration of chimeric antigen receptor-T (CAR-T) membrane coated nanoparticles for tumor photothermal therapy. CAR-T: chimeric antigen receptor-T; CIM: CAR-T cell membrane-coated nanoparticle; GPC3: Glypican-3; IR780: IR780 iodide; IM: IR780-loaded MSN; MSN: mesoporous silica nanoparticle; ScFv: single-chain variable region (derived from monoclonal antibody heavy and light chains and expressed on the cell membrane of CAR-T cells). Reproduced with permission from reference (Ma et al., 2020). Copyright ^©^ The author(s).

Recently, some newly emerged biological techniques have been used for the modification of cell membranes for anticancer agents loaded NPs camouflaging and/or therapy, which can not only camouflage the core NPs but be able to transfer engineered functionalized proteins, peptides and/or motifs to the tumor sites for treatment, and improve therapeutic efficacy (Jiang et al., 2020a; Bhattacharyya and Ghosh, 2020; Ma et al., 2020; Zinger et al., 2021). Ma et al. (Ma et al., 2020) fabricated a novel kind of NPs for hepatocellular carcinoma PTT (Figure 4). Mesoporous silica NPs loaded with IR780 were constructed as the core NPs, the cell membranes used for coating were derived from genetically engineered chimeric antigen receptors (CARs) T lymphatic cell and natural T lymphatic cell that extracted from human T cells enriched from peripheral blood mononuclear cells. Further studies *in vitro* and *in vivo* showed the CAR-T cells could recognize GPC3 expressed on the surface of hepatocellular carcinoma cells, which enhanced targeting abilities of the NPs; and the CAR T cells could eliminate cancer cells by single-chain variable region (ScFv) on the cell membranes of CAR-T cells, in a non-major histocompatibility complex-restricted way. These NPs showed superior biocompatibility, outstanding targeting ability and excellent photothermal response.

### 4.7 Cancer cell membrane‐camouflaged NPs

NPs coated with cancer cell membranes effectively deliver tumor-associated membrane-bound antigens with immunological adjuvants to antigen-presenting cells by inducing the anticancer immune response (Jiang et al., 2019; Li et al., 2020; Chen et al., 2021a; Zhao et al., 2021a; Fan et al., 2021; Shen et al., 2021). NPs functionalized with cancer cell membranes aid homotypic targeting, which, in turn, facilitates internalization by source cells through self‐recognition; these NPs strongly resist the immune system thanks to their capacity to replicate surface antigenic diversity (Chen et al., 2021a; Zhao et al., 2021a; Fan et al., 2021; Shen et al., 2021).

There were copious studies of cancer cell membrane-camouflaging in literature. Among them, in a work by Li et al. (Li et al., 2020), a biomimetic system of AM-PP@ICG NPs for targeted 19 F magnetic resonance/photoacoustic/fluorescence imaging-guided PTT was fabricated. The core was AM-PP@ICGNPs, and A549 lung cancer cell membranes were used as camouflage. Subsequent study indicated that the NPs had biocompatibility, time-dependent tumor homing with high sensitivity, a prominent photothermal effect for tumors under NIR laser irradiation, and a strong antitumor response *in vivo*. In another study by Zhao et al. (Zhao et al., 2021a), for the therapy of non-Hodgkin’s lymphoma, CCM@MSNs-ISOIM NPs were fabricated. In the process, mesoporous silica was used to load an herb-derived traditional Chinese medicine to form core NPs, and OCI-LY10 tumor cell membranes were used for camouflaging. The NPs exhibited good effects on blocking the lymphoma cell cycle and promoting mitochondrial-mediated apoptosis, with features of low pH sensitivity, high immune evasion, good biocompatibility, and active tumor targeting.

In previous research, the accumulation of payload delivered by cancer cell membrane-coated NPs in the tumor increased, and the mechanisms were that specific homotypic targeting and passive targeting via the EPR effect facilitated the delivery (Li et al., 2020). The core NPs were ICG loaded PLGA NPs, cancer cell membranes camouflaging facilitated effective targeting delivery, and these NPs demonstrated well in dual-modal (fluorescence/photoacoustic) image-guided PTT (Li et al., 2020). Yet, treatment resistance of the tumors to PDT owing to oxygen deficiency largely compromised the therapeutic efficacy; looking ahead, if oxygen levels in tumors can be increased, the therapeutic efficacy may be improved.

To solve the above issues, in further work, Chen et al. (Chen et al., 2021a) fabricated N/P@MCC NPs for PTT and PDT of tumor. Nitrogen-doped graphene quantum dots (N-GQDs) and protoporphyrin IX (PpIX) were constructed, with a hydrophobic mesoporous silica NP (HMSN) coating and loaded with catalase to obtain core NPs, and cancer cell membranes (CCMs) were used to coat core NPs via coextrusion. In the process of therapy, the N/P@MCC NPs generated ^1^O_2_, and yielded local low-temperature hyperthermia for thermally ablating cancer cells and infrared imaging. These NPs effectively scavenged excess H_2_O_2_ to sustainably maintain oxygen for synchronous O_2_ self-supply and hypoxia alleviation by augmenting the intrinsic catalytic features of catalase. Synergistically, the increased O_2_ reacted with N-GQDs and PpIX to enable a maximally increased ^1^O_2_ output for augmented PDT, thus facilitating hypoxic tumor elimination.

With the same consideration as above mentioned, Shen et al. (Shen et al., 2021) developed Ir–B–TiO_2_@CCM NPs for targeted cancer imaging and synergistic anticancer of PTT and SDT. In the process, B–TiO_2_ was prepared using chemical methods and functionalized with Ir to obtain Ir–B–TiO_2_ core NPs, cancer cell membranes were isolated from source cells of human cervical carcinoma (HeLa), and finally the cell membrane vesicles (CCMs) were camouflaged onto the core NPs through extrusion. The Ir–B–TiO_2_@CCM NPs generated heat efficiently under irradiation, catalytically formed ROS under ultrasound radiation, demonstrated selective localization in the mitochondria, and preferentially accumulated in cancerous rather than non-cancerous cells, thereby exhibiting targeting. Under synergistic irradiation at 1,064 nm and ultrasound radiation, the Ir–B–TiO _2_ @CCM NPs showed imaging effect and the tumor was identified with a high spatial resolution, meanwhile, these NPs completely eradicated tumors in a mouse model.

**FIGURE 5:**
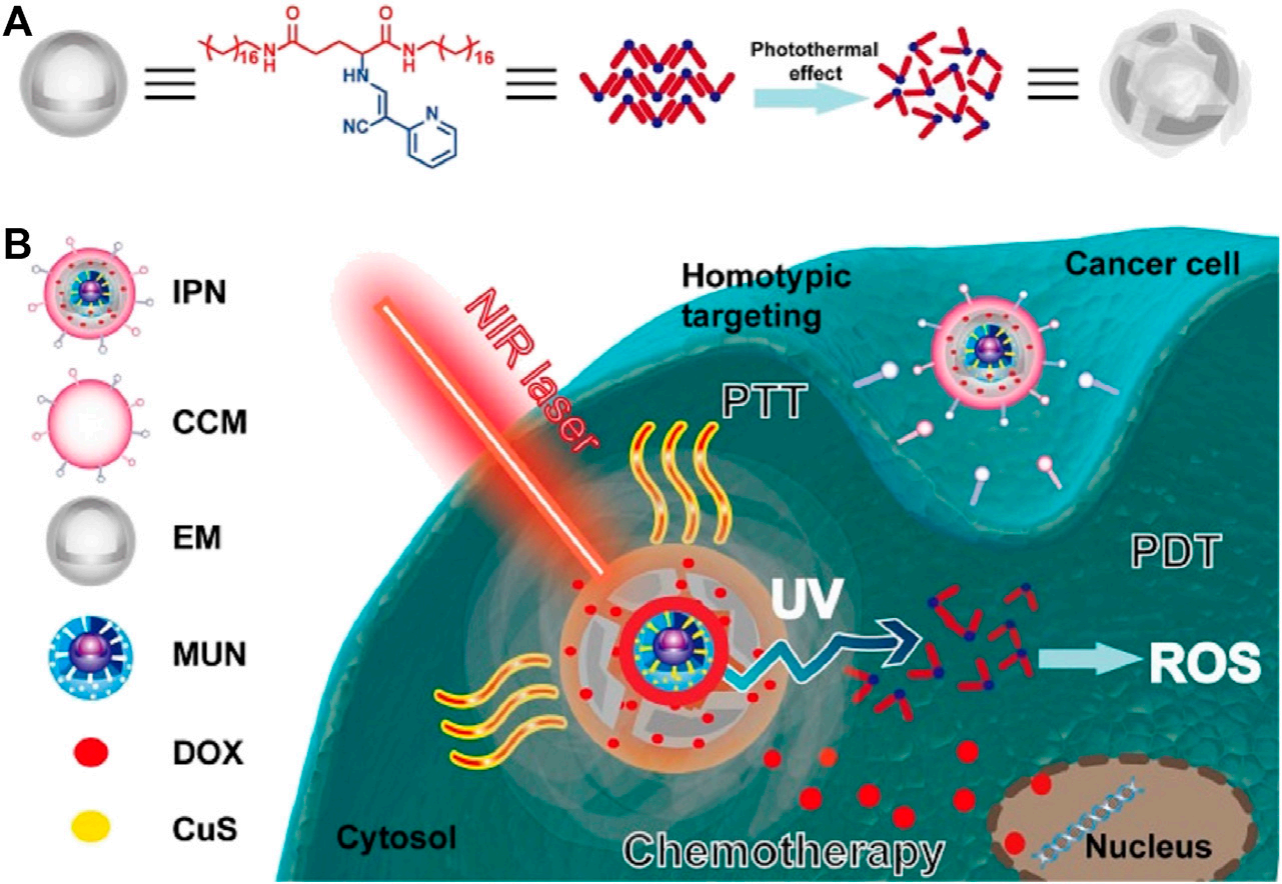
Schematic illustration of the intelligent phototriggered nanoparticles. **(A)** Chemical structure of EM gel and illustration of its photothermally induced phase transition; **(B)** Main components of IPNs and a schematic illustration of the domino effect induced by IPNs for multimodal tumor therapy. CCM: cancer cell membrane; CuS: copper sulfide; DOX: doxorubicin; EM: enaminitrile molecular; IPN: intelligent phototriggered nanoparticle; MUN: mesoporous silica-coated UCNP; NIR: near-infrared; PDT: photodynamic therapy; PTT: photothermal therapy; ROS: reactive oxygen species; UV: ultraviolet (UV); UCNP: upconversion nanoparticle. Reproduced with permission from reference (Xu et al., 2021). Copyright ^©^ The author(s).

To further improve therapeutic efficacy of biomimetic NPs, some new attempts were conducted. Xu et al. (Xu et al., 2021) developed intelligent phototriggered NPs (IPNs) for multimodal antitumor therapy of the breast cancer (Figure 5). They loaded mesoporous silica-coated upconversion (NaYF_4_: Yb/Tm) NPs (UCNPs) with copper sulfide to form core NPs, synthesized thermosensitive and photosensitive enaminitrile molecule (EM) organogel and loaded with antitumor drug DOX, encapsulated the core NPs with the antitumor drug DOX loaded EM organogel, and camouflaged them with cancer cell membranes isolated from breast cancer cells of MCF-7 and 4T1 cells for shell. The combined EM gel with UCNPs enabled PDT with NIR excitation, EM switched displaying H^+^-induced aggregate emission in tumor acidic microenvironments to generate ROS with ultraviolet irradiation, and was used as the second domino to control drug release. Subsequent studies showed that a domino effect was activated when irradiated the IPNs with an NIR laser, initiating photothermal effect by copper sulfide for PTT that also resulted in phase transformation of the anticancer DOX loaded EM gel to release DOX. In the process, the upconversion core converted the NIR light energy into ultraviolet light by exciting the EM, which generated ROS for PDT. The IPNs exhibited outstanding anticancer effects with little systemic toxicity.

### 4.8 Stem cell membrane-camouflaged NPs

Stem cell membrane-camouflaged NPs can evade immune system clearance, and thanks to their intrinsic tumor tropic property, are suitable for tumor therapy (Gao et al., 2016; Li et al., 2021b; Mu et al., 2021). Mu et al. (Mu et al., 2021) developed PDA-DOX/siPD-L1@SCM NPs to target prostate cancer (PCa) bone metastases. In their work, core NPs of PDA–DOX/siRNA were formulated using dopamine hydrochloride (polydopamine (PDA)), DOX, and siPD-L1 or siRNA^FAM^, and MSC membranes were camouflaged via coextrusion. Subsequent studies demonstrated that PDA-DOX/siPD-L1@SCM NPs effectively enhanced the blood circulation time and improved penetration ability and accumulation at tumor sites, and they exhibited excellent performance in the synergistic chemoimmunotherapy for PCa bone metastases.

In another study, Li et al. (Li et al., 2021b) fabricated DOX-loaded MSN@M NPs for antitumor treatment. In their work, core NPs were constructed by loading DOX with mesoporous silica (MSN), and sonication technique was used to camouflage the MSC derived membranes onto the core NPs. Ensuing study showed that DOX-loaded MSN@M NPs had high capacity for drug loading and sustained drug release, a reduced clearance rate, stronger tumor targeting and penetration abilities, more effective inhibition of tumor growth, and decreased side effects.

### 4.9 Hybrid cell membrane‐camouflaged NPs

In hybrid cell membranes cloaked NPs, multiple functionalities can be integrated into a single platform by fusing cell membranes isolated different cell sources, which can enhance the flexibility control of NP functionality and offer new opportunities for biomedical applications. For the effective treatment of breast cancer metastasis, Gong et al. (Gong et al., 2020) formulated DPLGA@[RAW-4T1] NPs. In their work, DOX-loaded PLGA (DPLGA) NPs were formulated as core NPs, cell membranes isolated from RAW264.7 macrophage cells (RAW) and 4T1 breast cancer cells (4T1) were combining used for fusion, and the sonication technique was used to coat the fused RAW-4T1 hybrid membranes onto the core DPLGA NPs. The hybrid membrane-coated DPLGA NPs exhibited excellent biocompatibility, targeted specific metastases, and displayed homogenous tumor-targeting ability *in vitro*. Beyond this, these NPs demonstrated a markedly enhanced multi-target ability in an animal model with lung metastases, and exhibited excellent anti-metastasis efficacy for the breast cancer-derived lung metastases.

In a further study, Jiang et al. (Jiang et al., 2019) constructed Melanin@RBC-M to enhance the PTT efficacy. In their work, melanin core NPs were extracted and formed from the ink sac of fresh cuttlefish and centrifuged. Hybrid membranes were created from RBC-derived and human breast cancer MCF-7 cell-derived membranes by cell membrane fusion. The hybrid membranes were coated onto melanin core NPs using an extrusion method. Ensuing study showed that the fused RBC-M hybrid membranes preserved cell membrane proteins of RBC and MCF-7 cell, and the resultant Melanin@RBC-M exhibited a prolonged blood circulation time, enhanced homotypic targeting ability, reduced cellular uptake by macrophages, and significantly increased tumor accumulation and PTT efficacy.

Beyond this, Xiong et al. (Xiong et al., 2021) fabricated Fe_3_O_4_-ICG@IRM for ovarian cancer therapy. In their work, ICG loaded magnetic NPs (Fe_3_O_4_) were the core NPs, ID8 ovarian cancer cell-derived and RBC-derived membrane vesicles were fused to yield hybrids through membrane fusion, and coextrusion technique was used to coat the hybrid membranes onto Fe_3_O_4_-ICG core NPs. Ensuing study showed that the Fe_3_O_4_-ICG@IRM preserved cell membrane proteins of both ID8 and RBC cell and exhibited highly specific self-recognition of ID8 cells, prolonged circulation time, and activated specific immunity. The NPs demonstrated good efficacy of synergistic PTT and immunotherapy. The immunotherapy was resulted from the release of whole-cell tumor antigens by photothermal-induced tumor necrosis.

For synergistic PTT and chemotherapy against hepatocellular carcinoma, Ji et al. (Ji et al., 2020) fabricated sorafenib (SF) loaded cancer cell-macrophage hybrid membrane-coated NIR-responsive hollow copper sulfide (CuS) NPs with a surface modified with anti-VEGFR (CuS-SF@CMV NPs). These CuS-SF@CMV NPs expressed the characteristic membrane proteins of both cancer cells and macrophages, exhibited good tumor cell targeting, high capacity of drug loading, increased immune evasion, and selectively accumulated in cancer cells *in vitro* and tumors *in vivo*. The CuS-SF@CMV NPs exhibited synergistic PTT and chemotherapy in cancer cells under NIR irradiation, and with 94.3% inhibition of tumor growth in a tumor-bearing mouse model. After the tumor cells were killed by the initial PTT, sorafenib and the anti-VEGFR antibody sustained the anticancer effect through inhibiting tumor cell proliferation and angiogenesis.

## 5 Conclusions and future perspectives

In this review, we have discussed the progress of using nanotechnology towards the design, formulation, characterization, function, effect and potential application of cell membrane-coated NPs, and some problems have been analyzed. Development of cell membrane-modified biomimetic NPs has progressed greatly in recent years. Their key value lies in how they are recognized as their respective source cells, which can support their prolonged circulation time, immune evasion, exceptional biocompatibility, and homotypic targeting. More so, some engineered cell membranes have extra therapeutic functions. Thanks to these advantages, NPs are strong candidates for use in cancer therapies; researchers aim to apply them to improve the therapeutic outcomes and reduce the side effects of existing therapies, to make advances in targeted drug delivery, chemotherapy, PTT, PDT, SDT and (photo) immunotherapy.

Nevertheless, NPs have several limitations: lack of precision understanding of the molecular mechanisms; denaturation of cellular membrane proteins may trigger potential immune responses to endogenous antigens, and profound study is insufficient; biomimetic nanoparticle-based PTT/PDT have lower light penetration, oxygen dependence, and limited therapeutic efficacy; tumor-targeting, payload-loaded NPs have low utilization efficiency as the delivery and distribution of tumor-targeting NPs work through the EPR effect; the EPR effect can become saturated, and an aberrant EPR effect causes NPs to lose access to the target cells in deep tissues of the tumor, thus lowering the sustainability of their accumulation in tumor.

Previous studies focus mainly on short-term effects of NPs stability, positive therapeutic response and side effects, and deficiency in longer systemic study and heterogeneity-related study. To achieve clinical translation of biomimetic NPs and a delivery system, NPs long-term toxicity, overall stability, the eventual potential to scale-up should be studied, and a system showing adequate *in vivo* promise must be considered too. In the future, further modifying the isolated cell membranes—such as to eliminate denatured cell membrane proteins, add a double-membrane coating, modify cell membranes via biological engineering and incorporate novel fabrication techniques and therapeutics; and use of modeling and computational simulations to justify design of NPs—will better optimize the outcomes we can currently achieve and support the development of new strategies for cell membrane-camouflaging anticancer therapy.

## Abbreviations

CTL: cytotoxic T-lymphocyte; CXCR4, C-X-C motif chemokine receptor 4; DOX, doxorubicin; EPR, enhanced permeability and retention; ICG, indocyanine green; MHC, major histocompatibility complex; MSC, mesenchymal stem cell; NK, natural killer; NIR, near-infrared; NP, nanoparticle; ^1^O_2_, single oxygen; PDGF, platelet-derived growth factor; PDGFR, platelet-derived growth factor receptor; PTX, paclitaxel; PDT, photodynamic therapy; PEG, polyethylene glycol; PLGA, poly(Lactic-co-glycolic acid); PPy, polypyrrole; PTT, photothermal therapy; RBC, red blood cell; ROS, reactive oxygen species; SDF, stromal cell-derived factor-1; SDT, sonodynamic therapy; TAN, tumor-associated neutrophil; UCNP, upconversion nanoparticle; VEGF, vascular endothelial growth factor; VEGFR, vascular endothelial growth factor receptor.
